# Path integration from optic flow and the role of eye movements

**DOI:** 10.1038/s41598-026-56170-9

**Published:** 2026-06-07

**Authors:** Renate Reisenegger, Frank Bremmer

**Affiliations:** 1 Applied Physics and Neurophysics, University of Marburg, Karl-v-Frisch Str 8a, 35043 Marburg, Germany; 2 Center for Mind, Brain and Behavior, Universities of Marburg, Giessen, and Darmstadt, Hans-Meerwein-Str. 6, 35032 Marburg, Germany; 3 Department of Physiology, School of Biomedical Sciences, Faculty of Medicine, Nursing, and Health Sciences, Monash University, Melbourne, Australia

**Keywords:** Distance perception, Optic flow, Self-motion, Eye movements, Fixation, Path integration, Neuroscience, Psychology, Psychology

## Abstract

Optic flow provides critical visual information for self-motion perception, including direction (heading), speed, and travel distance (path integration). While eye movements are naturally elicited during optic flow, most lab-based studies of human path integration require central fixation, potentially altering perception. Here, we examined whether free eye movements improve distance discrimination compared with fixation, testing the hypothesis that oculomotor signals may support *oculomotor odometry*. The participants viewed two consecutive optic flow stimuli simulating forward self-motion across a ground plane and judged which interval represented the longer travel distance. Eye movement instructions varied across the four conditions, allowing either free-viewing or requiring fixation (still allowing for miniature slow and fast eye movements within a control window) during each interval. Behavioral accuracy was analyzed with generalized linear mixed-effects models, and eye movements were quantified either including or excluding saccades. As expected, free-viewing induced larger and faster saccades and higher smooth eye-movement speeds than fixation did. However, contrary to our hypothesis, fixation did not systematically impair distance discrimination. While cumulative eye-movement amplitudes scaled with travel distance as predicted, psychometric functions for perceived distance were similar across conditions. These findings, together with results from other recent studies, suggest that fixation-based paradigms do not impair the study of certain aspects of self-motion perception, including settings requiring stable gaze (e.g., EEG recordings).

**Supplementary Information:**

The online version contains supplementary material available at 10.1038/s41598-026-56170-9.

## Introduction

Whenever we walk or move around the world, we experience a characteristic visual input signal, called optic flow^1,2^. Optic flow allows us to determine in which direction we are moving (heading)^3,4^, how much distance we have traveled^5–7^, and, within certain limits^8^, how fast we are moving^9,10^ (for a recent review, see, e.g.,^11^). Optic flow is also accompanied by characteristic optokinetic-like eye movements: slow eye movements following the motion of the stimulus near the fovea^12^ and rapid (saccadic) eye movements, often directed toward the focus of expansion (FOE)^13–16^.

Eye movements are not only induced by optic flow but also impact the way we see and perceive^17^. Slow eye movements have been shown to modulate the perception of visual motion (for a review, see^18^:) as well as other visual (e.g., color:^19^) and nonvisual signals (e.g., time:^20^). Similarly, saccadic eye movements affect visual and nonvisual perception (for reviews, see^21–23^). Eye movements during self-motion have been investigated in several species, and they are suggested, among others, to serve stabilizing the visual image on the retina, allowing for high-resolution vision^24,25^ or, e.g., keeping the image of a prey in a specified region of the retina^26,27^.

Unless performed under natural conditions^28–30^, self-motion studies often require participants to fixate a target on a screen in front of them (for reviews, see, e.g.,^4,31^). Such fixation, however, comes at costs. Active fixation has been shown to influence the perception of vection, i.e., the vivid impression of self-motion despite being still^32^. Moreover, cortical activation differs depending on whether participants are required to fixate on a target or not^33^. In addition, most studies investigating self-motion perception in humans have focused on direction, i.e., heading. Another key aspect of self-motion and navigation, however, is travel distance (path integration). Furthermore, participants are typically required to fixate while visual stimuli are presented mimicking a (straight-forward) self-motion (e.g.,^5–7,34–36^). Moreover, there is evidence that eye movement patterns during reproduction of a traveled distance closely resemble those observed during the initial encoding phase, with eye position and velocity correlating on a trial-by-trial basis^14^.

Path integration has been studied not only in humans but also in other animals, most prominently in insects (for reviews, see, e.g.,^37,38^). For those species, a remarkable functional property, the odometer, which can be based on intrinsic (e.g., stride length) or extrinsic (visual) cues, has been described^39–42^. In an impressive experimental study on desert ants, Wittlinger and colleagues^39^ unequivocally demonstrated the importance of stride length for successful path integration. The travel distance was overestimated by the experimental animals walking on stilts and underestimated by the animals walking on stumps. In a follow-up study, Pfeffer and Wittlinger reported that passively transported animals could also successfully solve a path integration task by integrating visual optic flow alone^40^. Similarly, intrinsic (idiothetic) and extrinsic cues are also relevant for human path integration (e.g.,^43–46^). Surprisingly, however, given the large set of previous works on path integration and given their abundant occurrence in everyday living, the role of restricting eye movements during path integration has, to our knowledge, not been studied thus far. This is remarkable since the slow phases of the spontaneous eye movements in principle could support path integration. It is known that the gain of the slow phases typically is in the order of 0.5 with respect to the speed of visual motion around the fovea^4,12,47^. Accordingly, the accumulated distance of the slow phases across a given displacement would correlate with the distance of that very displacement. This, in fact, would be the oculomotor odometer. The eye movements induced by visual optical flow are very similar (or even identical) to optokinetic nystagmus, OKN, i.e. they are reflexive. Accordingly, the oculomotor odometer would come automatically and would not be dependent on any other behavioural task in everyday self-motion.

Here, in a lab-based study, we aimed to determine whether fixation, compared with free eye movements, has an impact on path integration by asking participants to discriminate between two consecutive visually simulated self-motion distances with different eye movement instructions (fixation vs free-viewing).

We hypothesized better accuracy during free-viewing than during fixation, considering that (1) free-viewing is a more “natural” setting, and (2) free-viewing would, in principle, allow the movement of one’s own eyes to be integrated while tracking (oculomotor odometry).

## Materials and methods

### Participants

Twelve naïve volunteers (5 females and 7 males, aged between 19 and 38 years, mean 27.4 years) with normal or corrected-to-normal vision and without any known neurological or psychiatric disorders participated in our study and were compensated with 10 € per hour. Prior to the experiment, they provided written informed consent. Our study was performed in accordance with the Declaration of Helsinki and was approved by the local Ethics Committee (Psychology Department, University of Marburg).

### Apparatus

The participants sat in a darkened and sound attenuated room. The stimuli were presented on a 24-inch computer monitor (VPixx Technologies Inc., Saint-Bruno, QC, Canada) 57 cm in front of them at eye level. The participants’ heads were stabilized by a chin rest. The display was 49.80° wide and 29.15° high and was set to a resolution of 1920 × 1080 pixels at a refresh rate of 120 Hz. Eye movements were recorded via an EyeLink 1000 system (SR Research, ON, Canada); calibration was performed with a 9-point grid with a maximum error during validation of 1°.

### Stimulus and task

**Fig. 1:**
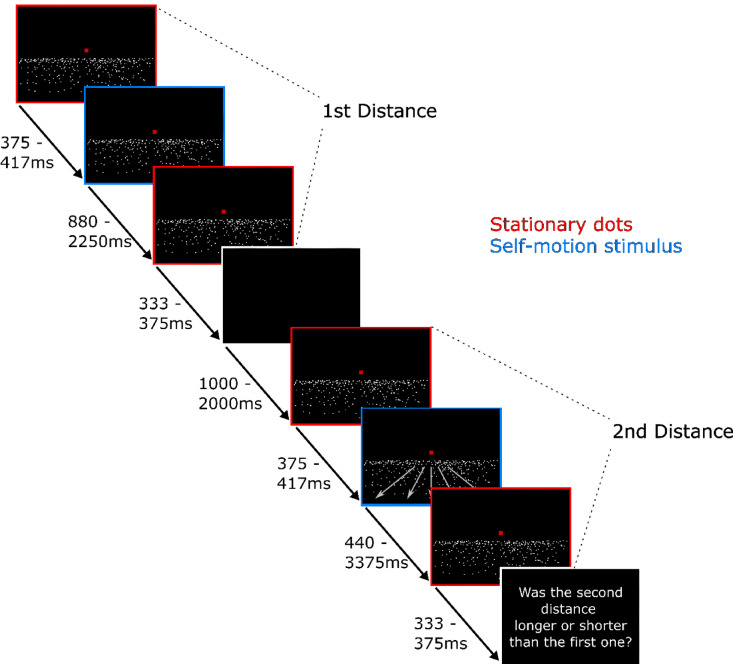
Experimental paradigm. The participants were shown simulated forward self-motion across a ground plane, preceded and followed by stationary intervals. Two such sequences were shown, separated by an intratrial interval (ITI. Black screen, random duration ranging uniformly from 1000 to 2000 ms across trials). The first self-motion stimulus (1st distance) consisted of three possible simulated displacements (70, 80 and 90 arbitrary units (AUs)) presented in pseudorandomized order and lasting between 880 and 2250 ms, depending on the simulated distance and speed (possible speeds: 20, 30 and 40 AU/s). The second self-motion stimulus (2nd distance) was determined as a pseudorandomized scaling factor (× 0.5, 0.7, 0.875, 1, 1.125, 1.3, 1.5) applied to the 1st distance and lasted between 440 and 3375 ms. At the end of the trial, participants had to indicate by a button press if they had perceived the 2nd distance to be longer or shorter than the first distance.

As shown in Fig. 1, we presented two consecutive optic flow stimuli that simulated self-motion across a ground plane with heading directed forward (straight ahead). The stimulus was implemented via MATLAB R2012a (The MathWorks. Inc., Natick, USA) and the psychophysics toolbox^48–50^.

The ground plane stimulus was composed of 2000 white dots with unlimited lifetimes on a black background. It covered the full horizontal width of the screen (central 49.80° of the visual field) and 13.37° in the lower visual field (starting 1.21° below the fovea). The dot size increased when approaching the virtual observer, ranging from a 1-pixel radius at the farthest distance to a 30-pixel radius when it was closest to the observer. When the distance to the participant was closer than the viewing distance (when they should have been displayed between the screen and the participant), the dots were repositioned to another random location of the ground plane.

For each trial, before and after each self-motion stimulus, there were short periods without movement (375–417 ms and 333–375 ms, respectively), i.e., ground plane dots were displayed stationary. In the first self-motion epoch resulting in a given displacement (“1st distance”), the motion showed 3 possible simulated distances (70, 80 or 90 arbitrary units (AUs)), with one of those distances being chosen pseudorandomly for each block and being presented for the whole block of 14 trials. For the second self-motion epoch (“2nd distance”), the simulated distances were a pseudorandom factor (from now on “distance ratio”, of 0.5, 0.7, 0.875, 1, 1.125, 1.3 or 1.5) multiplied by the 1st distance, covering all distance ratios twice throughout a given block. Each self-motion stimulus was presented with a pseudorandomized velocity of 20, 30 or 40 AU/s, resulting in motions taking 1750–4500 ms for the 1st distance and 875–6750 ms for the 2nd distance. The eye height was set at 2.67 AU, and the maximum simulated visibility (i.e., the maximum distance that the observer could see from the ground plane) was ~ 125 AU.

For each block, one of four eye movement conditions (fixation conditions)—Fix00, Fix01, Fix10, or Fix11—was assigned pseudorandomly. These labels indicate whether participants were required to fixate or were allowed to freely move their eyes during the first and second motion distances. Specifically, a “0” denotes free viewing (i.e., no fixation target shown), and a “1” denotes the presence of a central fixation target (ABC-target from^51^, with an outer radius of 0.16° and an inner radius of 0.04°). Accordingly, in Fix00, both distances were presented under free-viewing instructions. In Fix01, the first distance was free-viewing, and the second required fixation; in Fix10, the first distance required fixation, and the second was free-viewing; and in Fix11, both intervals required fixation. The assigned fixation condition remained constant throughout the duration of a block.

The participants were asked to observe the simulated self-motion distances and at the end of each trial answer if the second distance was longer or shorter than the first distance by pressing one of two keys on the keyboard. They were asked to fixate on the fixation target whenever it appeared on the screen. Trials containing blinks or unsuccessful fixations (eye positions outside of a control window centered on the fixation target) were immediately stopped and repeated from the beginning of the trial. The size of the control window was a circle of 2° radius (3° or 3.5° for two participants, due to difficulties measuring eye position while using their glasses). These are comparably large values, which were chosen based on previous work. It is known from the literature that large-field visual motion evokes involuntary residual optokinetic (like) responses during fixation^52^, i.e., fixation reduces but cannot fully eliminate reflexive eye movements. Accordingly, we had to choose a window size that allows for the occurrence of such residual eye movements without aborting a trial, and at the same time keep their amplitude well outside the range of the free eye movement condition.

### Behavioral data analysis

For each distance ratio, participant-level accuracy scores were calculated and standardized via z-scores. Participants whose accuracy deviated more than ± 2.5 standard deviations from the mean were considered outliers (one participant) and excluded from all subsequent analyses.

For each participant and distance ratio, the mean accuracy was computed by averaging across trials. These participant-level means were then aggregated across participants to compute the group-level mean and standard deviation (SD) of accuracy for each distance ratio.

From our first hypothesis, we expected a reduced behavioral performance during fixation instructions compared with free-viewing instructions. To investigate this, we tested whether fixation conditions predicted perceptual accuracy on a trial-by-trial basis. We used a generalized linear mixed-effects model (GLMM) implemented via the *lme4* package in R^53^. GLMMs are particularly well suited for binary psychophysical data. Unlike approaches that rely on e.g. fitting psychometric functions separately per subject, GLMMs incorporate random effects to capture inter-individual variability in bias and sensitivity^54^. Thereby, they improve statistical power and enable direct inference about experimental factors and avoid possible distortions introduced e.g. by averaging^55^. The model was estimated via restricted maximum likelihood (REML), the BOBYQA optimizer and setting the family to binomial. First, we fitted a full model with Answer (i.e., whether the second distance was judged as longer) as the dependent variable and included fixation condition, distance ratio (z-scored), difference in speed and block number as fixed effects. All possible interactions with the fixation condition were included (formula: *Answer* ~ 1 + *fixation condition* * *distance ratio* + *fixation condition* * *difference in speed* + *fixation condition* * *block*). Using a stepwise backward selection procedure based on likelihood ratio tests, we removed noncontributory terms to arrive at the simplest model able to confidently fit our data. The final model retained only the fixation condition and distance ratio as fixed effects (formula: *Answer* ~ 1 + *fixation condition* + *distance ratio*). All the models included random intercepts and slopes for distance ratios for each participant (formula: ~ 1 + *distance ratio*|*ParticipantID*). Model summaries and tables were built via the *sjPlot* package^56^. Marginal and conditional R^2^ values were calculated via *sjPlot* based on the method described by Nakagawa et al.^57^. We assessed the goodness of fit of the GLMM via the *DHARMa* package^58^ in R, which provides simulation-based residual diagnostics. There was no over- or under-dispersion in the residuals (dispersion = 1.00, *p* = 0.94), suggesting that the variance of the residuals was consistent with the model assumptions. The proportion of observed zeros closely matched the expected number under the fitted model (observed-to-simulated ratio = 1.001, *p* = 0.97), indicating that zero-inflation was not present. Finally, a small number (< 1%) of residuals were flagged as potential outliers (33 out of 3696 trials), but this frequency was within the expected range (*p* = 0.52). Together, these results suggest that the model provides an adequate fit to the data.

To quantify perceptual performance, we derived the point of subjective equality (PSE) and the just noticeable difference (JND) from the fitted mixed-effects logistic regression model, including the interaction term between the fixation condition and distance ratio (*Answer* ~ 1 + *fixation condition* * *distance ratio* + *(*1 + *distance ratio*|*ParticipantID)*. For each fixation condition, the PSE was defined as the distance ratio at which the predicted probability of judging the second distance as being longer reached 0.5. This was computed as:

1 $$PSE = - \frac{{\beta_{0} + \beta_{fix} }}{{\beta_{1} + \beta_{fix \times slope} }}$$

where β_0_ is the model intercept, β_fix_ is the main effect of the fixation condition, β_1_ is the slope with respect to the distance ratio, and β_fix×slope_ is the interaction term.

The JND was defined as the difference in the stimulus value required to shift the response probability from 0.5 to 0.75. For logistic psychometric functions, this is given by:

2 $$JND = \frac{ln\left( 3 \right)}{{\beta_{1} + \beta_{fix \times slope} }}$$

We obtained standard errors and 95% confidence intervals for both the PSE and JND estimates via the delta method, which propagates uncertainty from the covariance matrix of the fixed-effect estimates. To facilitate interpretation, estimates were transformed back to the original units of the distance ratio (i.e., reversing the z-scoring of the predictor).

Finally, to assess whether fixation conditions differed in PSE, we conducted pairwise comparisons between all fixation levels. Differences were tested using z-tests based on the independent standard errors of the PSE estimates, and the resulting *p*-values were corrected for multiple comparisons via the Bonferroni method.

For visualization, we used the predictions of the fixed effects of the same GLMM fitted with the interaction term. Confidence intervals (95%) for the predicted probabilities were calculated on the logit scale and back-transformed to the probability scale.

To ensure that our estimates of the PSE and JND were not biased by stimulus-independent lapses, we performed a secondary robustness check. While our GLMM accounts for intercept and slope variance, it does not explicitly parameterize lapses. We therefore refit the data using a Bayesian non-linear mixed-effects model (using the *brms*^59^ package in R) that explicitly estimates the participant-specific lapse rates (λ). This model uses a non-linear link function where the probability of the responses is constrained by λ at the floor and ceiling of the psychometric function. Comparison between the GLMM and the Bayesian models revealed slight shifts in the JND parameters, but the relative differences between the conditions remained unchanged (See Supplementary Materials for full model specification and comparison tables).

All summary statistics, models and figures were computed in RStudio (v2025.05.1)^60^ with R (v4.5.0)^61^ via *dplyr*^62^, emmeans^63^ and *ggplot2*^64^ in addition to the previously mentioned packages.

### Eye movement data preprocessing and analysis

We low-pass filtered the eye-position data via a second-order Butterworth filter with a cutoff frequency of 15 Hz. Eye velocity, obtained by differentiating the filtered eye position data, was low-pass filtered via a second-order Butterworth filter with a cutoff frequency of 30 Hz. Saccades were detected using a velocity threshold of 10°/s for a minimum duration of 5 ms. The nearest reversal in the sign of the acceleration, obtained by differentiating the filtered velocity, determined saccades on and offsets. To characterize the saccadic events associated with the different eye-movement instructions, we computed and analyzed the saccade amplitude and peak velocity. The group data for peak velocity and amplitude were confirmed to be normally distributed for both fixation instructions via a Lilliefors test (*p* > 0.05). A paired *t*-test was used to determine significant differences between instructions with a threshold of *p* < 0.05 for significance.

The vertical and horizontal eye movement speeds were calculated as the sum of the absolute value of the difference in the vertical and horizontal filtered positions, respectively, divided by the total time of that trace. This was done with the complete eye movement trace from the onset until offset of the self-motion stimulus as well as traces after saccade deletion (slow eye movements, SEM). To assess the impact of eye-movement instructions on eye-movements, we conducted paired-sample *t*-tests on the data averaged by participant. Specifically, we compared the horizontal and vertical components under our two eye-movement instructions (free-viewing vs. fixation), in addition to comparing how the fixation instructions influenced the horizontal and vertical speeds separately. To correct for multiple comparisons, *p*-values were Bonferroni-corrected.

All eye-movement data preprocessing and transformations and paired-sampled *t*-tests were conducted using customized programs developed in MATLAB R2022a (The MathWorks. Inc., Natick, USA).

The following modeling was performed on slow eye movements (SEMs) because a previous study suggested the importance of SEMs in self-motion tasks^14^ and that fast eye movements compromise the encoding of visually simulated self-motion^65^.

From our second hypothesis, we expected the SEMs to be predicted by the self-motion distance (a greater travel distance resulting in more slow eye movements). To test this, we fitted a simple linear mixed-effects model (LMM) via the *lme4* package in R^53^ (estimated via restricted maximum likelihood (REML) and the BOBYQA optimizer) to predict SEMs with only fixation instructions and traveled distance as fixed effects (formula: *SEMs* ~ 1 + *fixation instructions* * *traveled distance*), with ParticipantID as a random effect (formula: ~ 1 | *ParticipantID*). Using a stepwise forward selection procedure based on likelihood ratio tests, we added fixed and random effects (and their interactions), retaining terms that significantly improved the model fit. The final model included the interaction between eye-movement instruction with traveled distance and with block number, in addition to the interaction of travel distance and travel speed (and the individual terms) as fixed effects (formula: *SEM* ~ 1 + *fixation instructions* * *travel distance* + *speed* * *travel distance* + *fixation instructions* * *block* + *distance number*). The model included random intercepts and slopes for travel speed and distance for each participant (formula: 1 + *speed* + *travel distance* | *ParticipantID*).

The LMM results tables were built via the *sjPlot* package^56^, which computes *p*-values based on conditional *F*-tests with a Kenward-Roger approximation via the *pbkrtest* package in R^66^. Marginal and conditional R^2^ values were calculated *sjPlot* based on^57^.

ChatGPT^67^ was used to assist with debugging for the analyses code and scripts for figure generation.

### Code availability

Stimulus and analysis scripts together with the raw and aggregated data can be found at https://tam-datahub.online.uni-marburg.de/handle/tam/43, with README files for the overall structure of the repository and instructions for the analysis pipeline.

## Results

### Dependence of fixation conditions on perceptual accuracy

In a given trial, participants were shown two self-motion sequences and had to judge at the end of the trial if the travel distance of the second self-motion was longer or shorter than that of the first. The participants had to solve this discrimination task with two possible eye-movement instructions for each distance, resulting in 4 different fixation conditions: Fix00, Fix01, Fix10 and Fix11 (where “1” denotes required fixation and “0” indicates free-viewing; for details, see Materials and methods). During free-viewing, participants were free to move their gaze, and during fixation, they had to fixate on a central target. Fixation was monitored online, and for a trial to be considered valid, the eye position had to be within a control window centered on the target throughout a given trial.

**Fig. 2:**
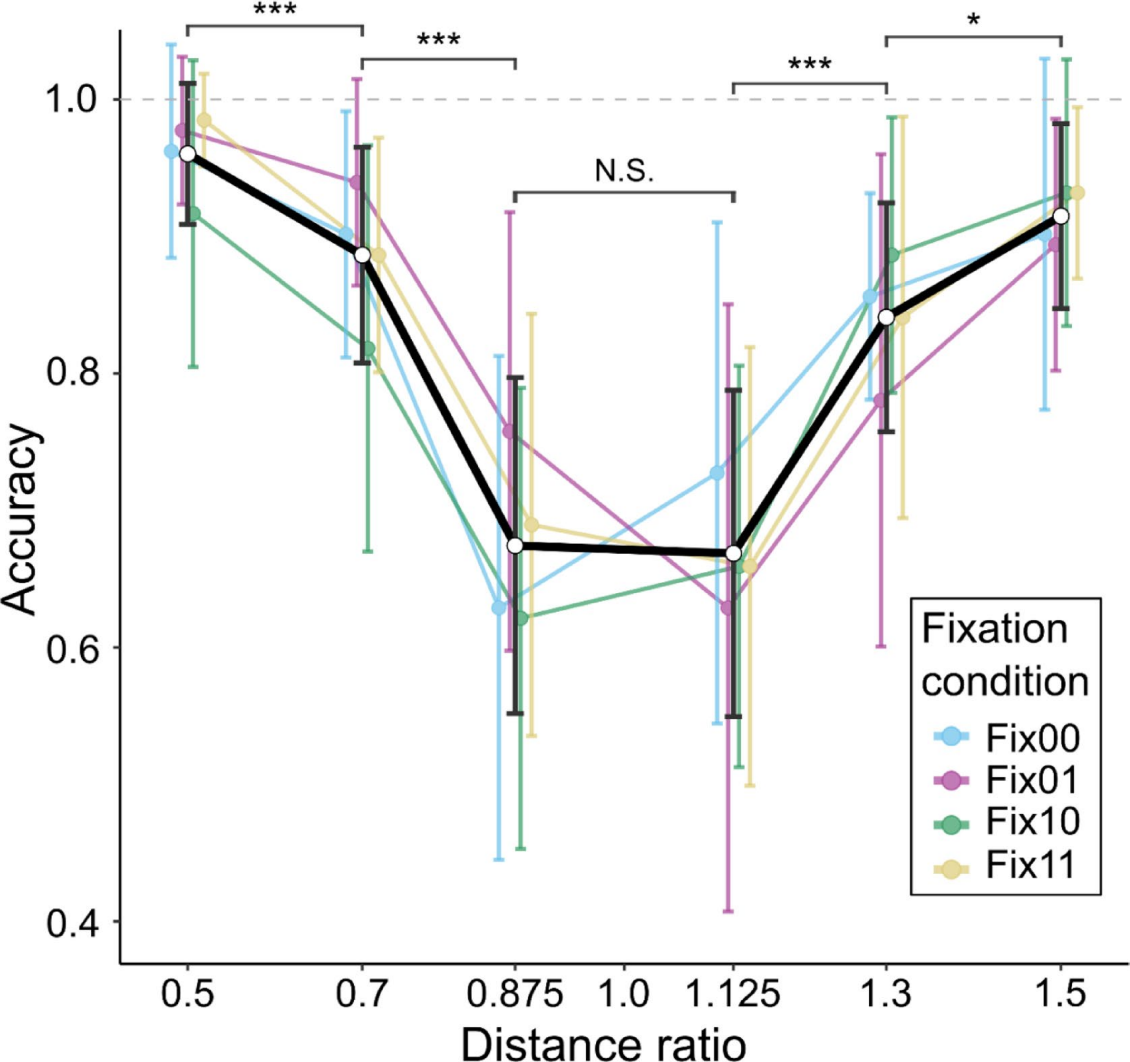
Accuracy as a function of the distance ratio. Each colored dot depicts the mean values across all participants for a given condition, and each white dot depicts the mean value for all participants and conditions (values for the distance ratio are identical for each condition; data points and associated error bars were horizontally displaced only for presentation). The error bars represent one standard deviation (SD). Asterisks indicate the results of a Wilcoxon test with multiple comparison correction for the grouped results (***: *p* < 0.001, *: *p* < 0.05, N.S.: *p* > 0.05).

To evaluate how these fixation conditions affected perceptual judgments, we analyzed participants’ accuracy as a function of the distance ratio, which was defined as the 2nd distance/1st distance. Each individual fixation condition, as well as the group-level mean accuracy, presented the lowest values close to the distance ratio of 1 and increased for lower and higher distance ratios, as shown in Fig. 2. This was expected, given that lower and higher distance ratios indicate that the distances were more different from each other and therefore easier to discriminate.

We fitted a generalized linear mixed-effects model (GLMM) to predict participants’ responses (i.e., whether the second distance was judged as longer) using the fixation condition and the z-scored distance ratio as fixed effects, with random intercepts for ParticipantID. The model revealed a significant effect of fixation condition (Χ^2^(3) = 26.53, *p* < 0.001) and distance ratio (OR = 7.53, 95% confidence interval (CI) [5.44, 10.42], *p* < 0.001) (see Table 1). Specifically, compared with the baseline fixation condition (Fix00, free-viewing during both self-motion epochs), the Fix01 condition (i.e., free-viewing during the first and fixation during the second self-motion epoch) was associated with significantly lower odds of judging the second distance as longer (OR = 0.61, 95% CI [0.48, 0.78], *p* < 0.001), which is in line with the idea of an oculomotor-odometer, where restricting eye-movements would lead to an under-estimation of the perceived self-motion distance because participants would not be able to use oculomotor tracking of the stimulus. On the other hand, the Fix10 and Fix11 conditions did not significantly differ from the baseline condition Fix00 (Fix10: OR = 1.10, 95% CI [0.86, 1.39], *p* = 0.458; Fix11: OR = 0.81, 95% CI [0.64, 1.04], *p* = 0.095). In addition, the interaction between the fixation condition and the distance ratio did not improve our model significantly (Χ^2^(3) = 3.18, *p* = 0.365), which contradicts our predictions that fixation instructions impair self-motion distance perception.

**Table 1:** Generalized linear mixed-effects model results for predicting answers.

	Answer		
Predictors	Odds ratios	CI	*p*
(Intercept)	1.04	0.75 – 1.43	0.824
Fixation [Fix01]	0.61	0.48 – 0.78	**< 0.001**
Fixation [Fix10]	1.10	0.86 – 1.39	0.458
Fixation [Fix11]	0.81	0.64 – 1.04	0.095
Distance ratio z	7.53	5.44 – 10.42	**< 0.001**
Random effects			
σ^2^	3.29		
τ_00_ _SubjectID_	0.21		
τ_11_ _SubjectID.Distance_ratio_z_	0.25		
ρ_01_ _SubjectID_	− 0.32		
ICC	0.12		
N _SubjectID_	11		
Observations	3696		
Marginal R^2^ / Conditional R^2^	0.524/0.582		

The results of our generalized linear mixed-effects model with the formula: *Answer* ~ *1* + *Fixation* + *Distance_ratio_z* + (1 + *Distance_ratio_z* |*ParticipantID*)). σ^2^: Random effects (within-participant) variance. τ_00_ _ParticipantID_: between-participant variance. τ_11_ _SubjectID.Distance_ratio_z_: between-participant slope variance. ICC: Intraclass correlation coefficient or proportion of the variance explained by random effects. N _ParticipantID_: number of participants. Observations: number of trials. Marginal R^2^: proportion of variance explained by the fixed effects. Conditional R^2^: proportion of variance explained by the model. Significant *p*-values (*p<*0.05) are shown in bold.

**Fig. 3:**
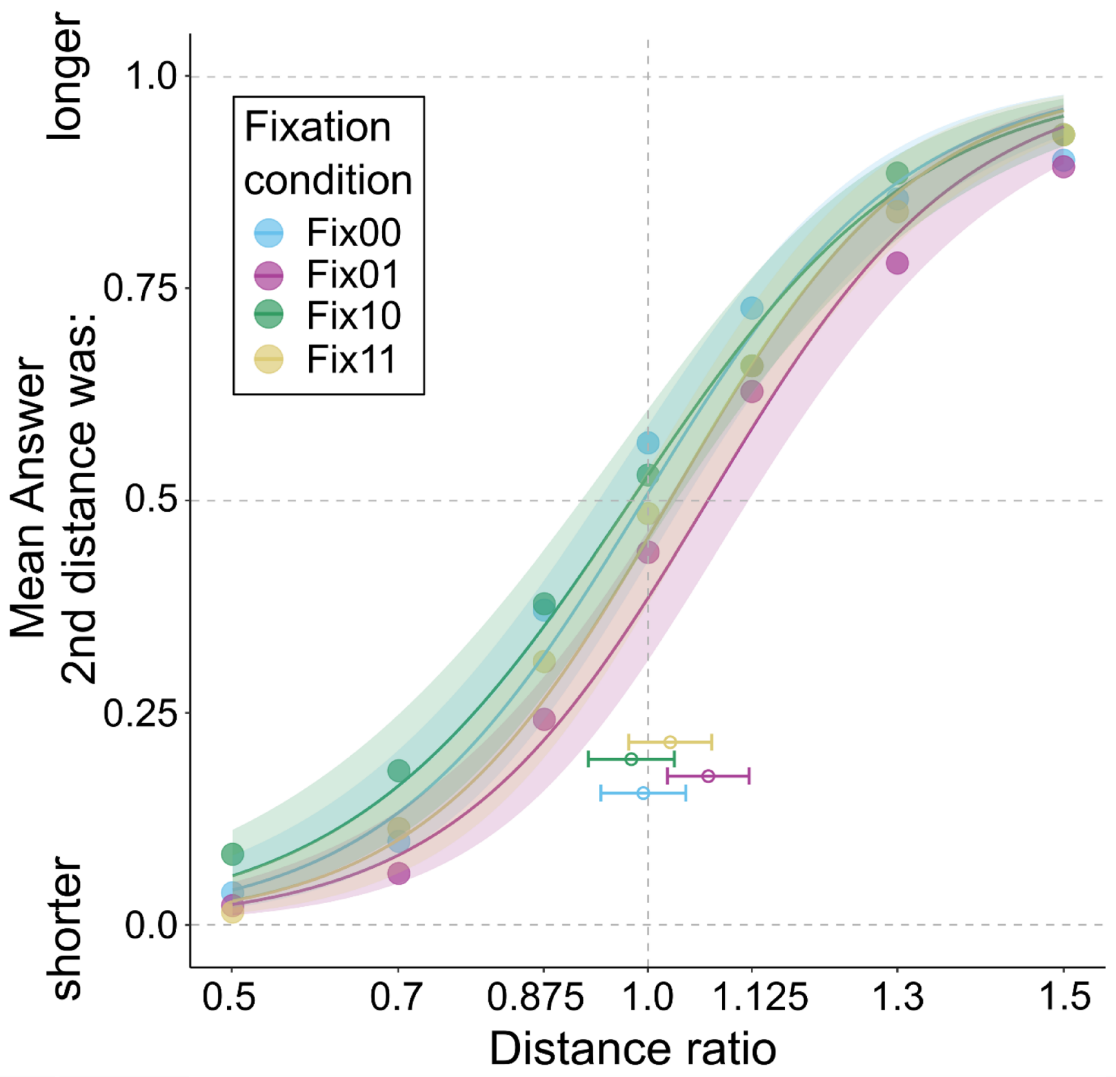
Generalized linear mixed-effects model predictions for all fixation conditions. The mean proportion of “2nd distance was longer” responses for each fixation condition (Fix00: blue, Fix01: pink, Fix10: green, Fix11: yellow) are shown as a function of the distance ratio (filled dots), superimposed with the model-predicted psychometric functions (solid lines) and their 95% confidence intervals (shaded areas). Empty dots with horizontal error bars depict the point of subjective equality (PSE) and the 95% CI for each fixation condition.

From the psychometric fits, we obtained estimates of the point of subjective equality (PSE) and the just noticeable difference (JND) for each fixation condition. PSEs were centered close to the physical equality point, indicating that overall judgments were largely unbiased across conditions. Pairwise comparisons revealed no significant differences in PSE between fixation conditions (p_adj_ ≥ 0.085, Bonferroni-corrected), suggesting only small shifts in perceived equality between different conditions. The minimum PSE was observed for the Fix10 condition, which led to a slight overestimation of the second distance compared with the first distance (PSE = 0.98). The opposite was found for the Fix01 condition with a slight underestimation of the second distance compared with the first (PSE = 1.07). For the JNDs, which reflect response precision, estimates were comparable across fixation conditions, and no pairwise differences reached significance after correction, in accordance with our GLMM selection procedure based on likelihood ratio tests where an interaction of fixation condition and distance ratio would not improve the model fit. This finding indicates that fixation did not strongly influence sensitivity to differences in the distance ratio. Figure 3 shows the mean proportion of “2nd distance was longer” responses for each fixation condition as a function of the distance ratio, overlaid with the model-predicted psychometric functions and their 95% CIs, as well as the estimates for the PSEs and their 95% CIs.

### Eye movements across different eye movement instructions and fixation conditions

We monitored participants’ oculomotor behavior online with an eye position control window of 2 deg radius centered on the fixation target. This diameter is necessary since even during fixation, the eyes are never completely still^68,69^. Hence, we also expected some residual eye movements during fixation. However, our experimental idea required these eye movements to be significantly smaller than they were during free gaze. To validate this key premise, we characterized saccade parameters as well as eye-movement speed (for the full eye-movement traces and for the saccade-free traces) for free-viewing versus fixation instructions.

**Fig. 4:**
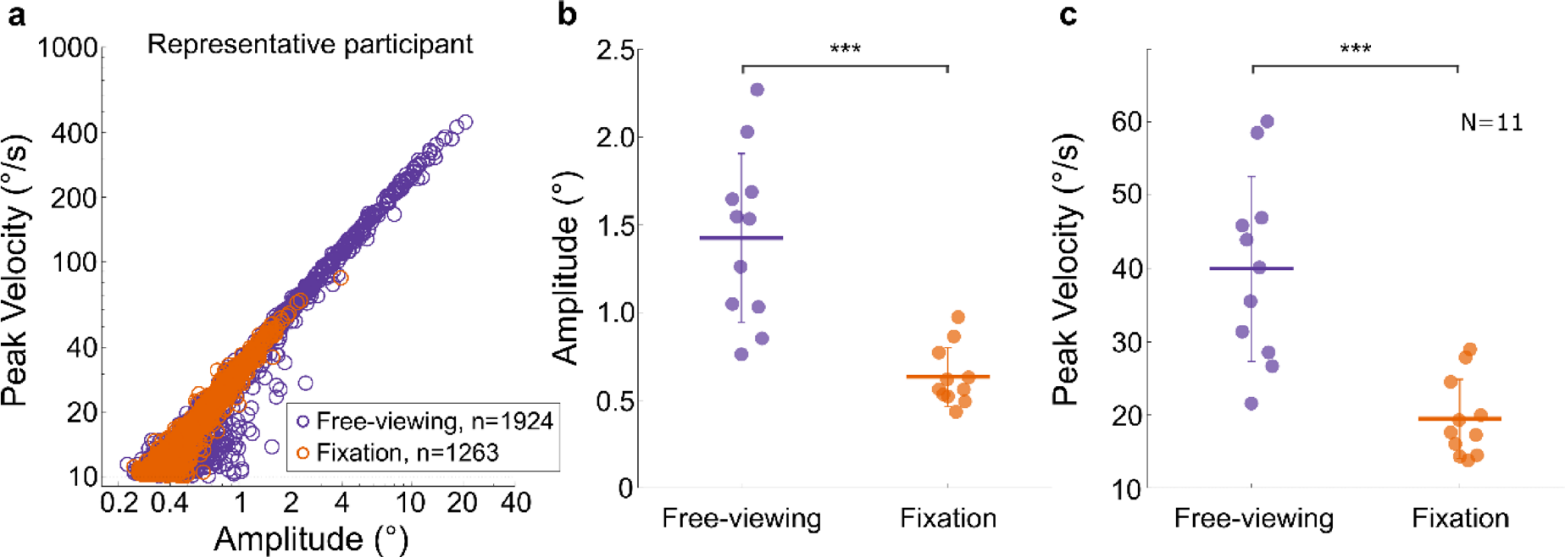
Saccade characteristics for a representative participant (**a**) and across participants (**b**, **c**). (**a**) Peak velocity as a function of amplitude (main sequence) of saccades shown on a log‒log scale. Each circle represents one saccade with color indicating the fixation condition (purple: free-viewing; orange: fixation). (**b**), (**c**) Each circle represents the mean peak velocity and amplitude from a given participant. The horizontal lines show the means across all participants with their standard deviations indicated by vertical error bars, each for the respective fixation condition (colors as in a: purple: free-viewing; orange: fixation). Asterisks indicate the *p*-value of a paired *t*-test (***: *p* < 0.001).

For the saccades, we found a linear relationship between peak velocity and amplitude (main sequence^70^) during free-viewing and fixation, with smaller saccades mostly corresponding to the fixation instruction (Fig. 4a). This result is in line with data previously shown by Chow and colleagues^71^ in a similar task. A paired-samples *t*-test indicated that saccade amplitudes and peak velocities were significantly greater in the free-viewing condition (mean(Amp) = 1.42°, SD(Amp) = 0.48°, mean(Vel) = 39.93°/s, SD(Vel) = 12.6°/s) than in the fixation condition (mean(Amp) = 0.63°, SD(Amp) = 0.17°, mean(Vel) = 19.46°/s, SD(Vel) = 5.4°/s) (amplitude: *t*(10) = 5.95, *p* < 0.001; peak velocity: *t*(10) = 6.28, *p* < 0.001) (Fig. 4b, c). In summary, as expected and intended, participants made larger and faster saccades when allowed to freely view the stimuli than when they were required to keep their eyes still.

**Fig. 5:**
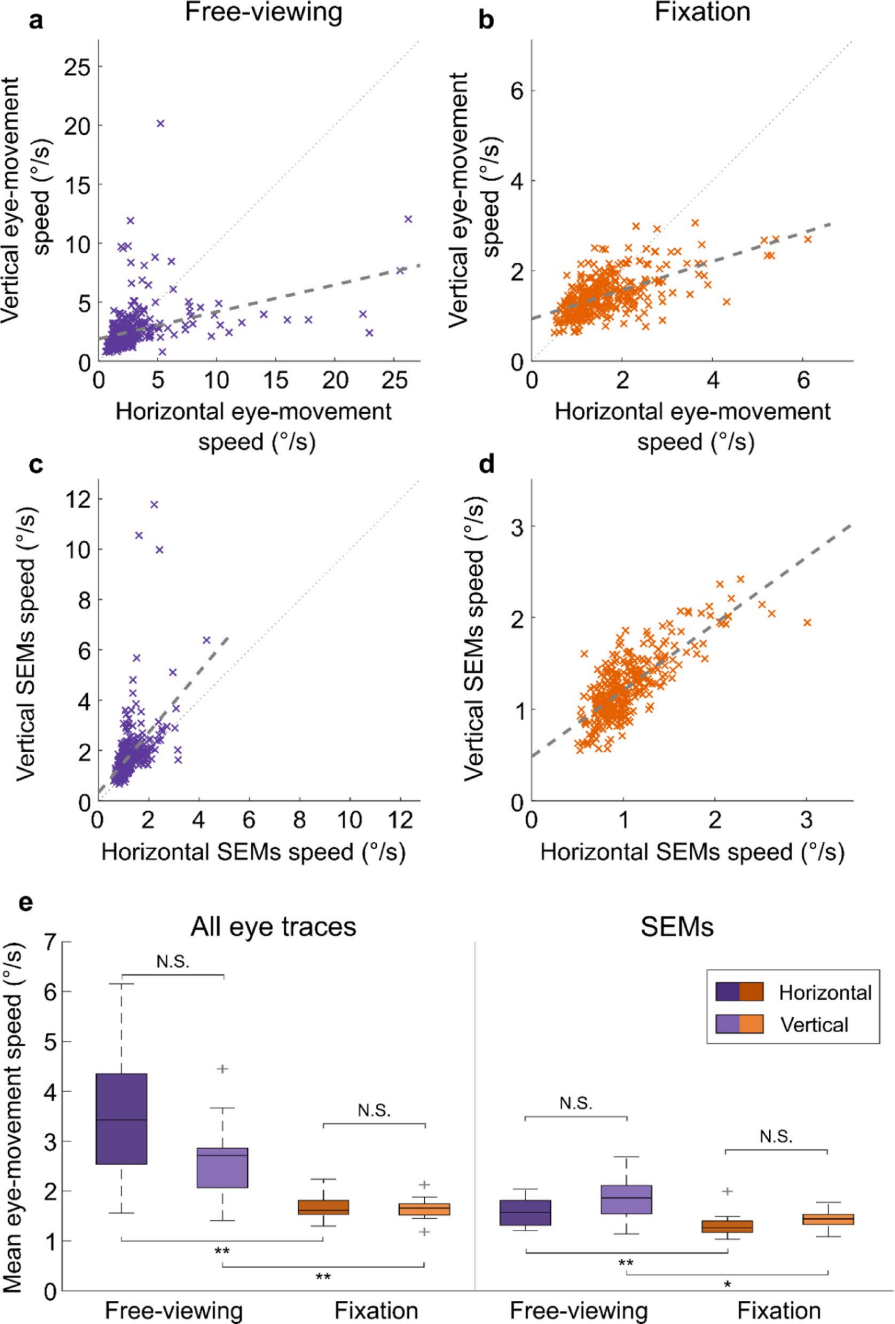
Vertical and horizontal eye movement speed with and without saccades for a representative participant (**a**–**d**) and group data (**e**). (**a**, **b**) Eye movement speed considering all eye movement traces (including saccades). (**c**, **d**) Slow eye movement (SEM) speed only (excluding saccades). (**a**–**d**) Each data point shows the eye-movement speed during free-viewing (**a**, **c**) or fixation (**b**, **d**) instructions for one self-motion distance for a representative participant. The gray dashed lines represent the linear regression. (**e**) Boxplots of mean eye-movement speed per participant across eye-movement instructions. The horizontal black line inside each box represents the median; the top and bottom edges indicate the third and first quartiles, respectively. Whiskers extend to data points within 1.5 times the interquartile range from the box limits. Individual points beyond the whiskers are plotted as outliers (gray crosses). Asterisks indicate the *p* value of a paired *t*-test after multiple comparison correction (**: *p* < 0.01, *: *p* < 0.05). (**a**–**e**) Free-viewing is depicted in purple, fixation in orange; (**e**) Darker colored boxes represent horizontal speed, and lighter colored boxes represent vertical speed.

We conducted paired-sample *t*-tests to compare eye-movement speed. The mean horizontal and vertical eye-movement speeds were compared between the free-viewing and the fixation instructions. We performed this analysis for all eye traces and for (de-saccaded) smooth eye movements (SEMs) only. We also analyzed how horizontal or vertical eye-movement velocities alone were affected by our eye-movement instructions (see Fig. 5). We observed the expected behavior: participants exhibited greater eye movement speed during free-viewing than during fixation for all comparisons (All eye-movements: horizontal: *t*(10) = 4.73, *p* = 0.0064; vertical: *t*(10) = 4.88, *p* = 0.0051. SEMs: horizontal: *t*(10) = 4.5, *p* = 0.0092; vertical: *t*(10) = 4.05, *p* = 0.0187). Interestingly, when we compared horizontal speed with vertical speed within a given instruction, we found no significant differences (all eye-movements: free-viewing:* t*(10) = 3.11, *p* = 0.0883; fixation: *t*(10) = 0.53, *p* = 0.1905; SEMs: free-viewing: *t*(10) = − 2.66, *p* = 1.0; fixation: *t*(10) = − 2.12, *p* = 0.4816).

### Dependence of slow eye movements on discrimination of distances

We fitted a linear mixed-effects model (LMM) to predict participants’ SEMs from the experimental conditions (see Materials and methods). The model revealed several significant predictors of the SEMs (see Table 2). Distance had a strong positive effect on the SEMs (β = 0.33, 95% CI [0.18, 0.48], *p* < 0.001), indicating that the participants were more likely to move their eyes for greater distances. Conversely, the fixation instruction had a large negative main effect (β = − 0.40, 95% CI [− 0.48, − 0.32], *p* < 0.001), suggesting reduced SEMs when the fixation target was present. These findings confirmed that our experimental conditions achieved the expected eye-movement outcome. Speed also negatively influenced pursuit behavior (β = − 0.28, 95% CI [− 0.44, − 0.11], p = 0.003), with faster speeds associated with decreased SEMs, which is explained by trials with slower speeds lasting longer than those with faster speeds (stimulus speed showed a strong positive effect on SEM speed: β = 0.23, 95% CI [0.21, 0.25], *p* < 0.001). In addition, the epoch (1st distance versus 2nd distance) had a small but reliable negative effect (*β* = –0.07, 95% CI [– 0.11, – 0.03], *p* < 0.001). The model revealed significant interactions between travel distance and fixation instruction (β = -0.09, 95% CI [− 0.13, − 0.06], *p* < 0.001) and between travel distance and stimulus speed (β = -0.08, 95% CI [− 0.09, − 0.06], *p* < 0.001), indicating that the effect of distance on SEMs was modulated by both fixation instruction and stimulus speed. Additionally, a small but significant fixation instruction with block interaction (β = 0.01, 95% CI [0.01, 0.02], *p* < 0.001) suggested adaptation effects across experimental blocks. The model accounted for substantial variance in SEM behavior (marginal R2 = 0.182, conditional R2 = 0.335), with considerable between-participant variability (ICC = 0.19).

**Table 2:** Results of the linear mixed-effects model predicting slow eye movements (SEMs).

	Slow eye movements (SEM)			
Predictors	Estimates	CI	*p*	df
(Intercept)	0.18	0.02 – 0.34	**0.033**	16.72
Distance	0.33	0.18 – 0.48	**< 0.001**	12.61
Fixation	− 0.40	− 0.48 – − 0.32	**< 0.001**	7371.64
Speed	− 0.28	− 0.44– − 0.11	**0.003**	12.10
Block	− 0.00	− 0.00 – 0.00	0.581	7371.73
DistanceNumber^2^	− 0.07	− 0.11 – − 0.03	**< 0.001**	7367.31
Distance × fixation	− 0.09	− 0.13 – − 0.06	**< 0.001**	7371.28
Distance × speed	− 0.08	− 0.09 – -0.06	**< 0.001**	7376.80
Fixation × block	0.01	0.01 – 0.02	**< 0.001**	7373.20
Random effects				
σ^2^	0.67			
τ_00_ _ParticipantID_	0.05			
τ_11_ _ParticipantID.Speed_	0.06			
τ_11_ _ParticipantID.Distance_	0.05			
ρ_01_	-0.25			
	0.25			
ICC	0.19			
N _ParticipantID_	11			
Observations	7392			
Marginal R^2^ /conditional R^2^	0.182/0.335			

The results of the linear mixed-effects model with the following formula: *SEM* ~ 1 + *fixation instructions* * *traveled distance* + *speed* * *traveled distance* + *fixation instructions* * *block* + *distance number* + (1 + *speed* + *traveled distance* | *participantID*)). σ^2^: Random effects (within-participant) variance. τ_00_ _ParticipantID_: between-participant variance. τ_11_ _ParticipantID.Speed_, τ_11_ _ParticipantID.Distance_: variance of random slopes for speed and distance across participants. ρ_01_: correlations between random effects. ICC: Intraclass correlation coefficient or proportion of the variance explained by random effects. N _ParticipantID_: number of participants. Observations: number of trials. Marginal R^2^: proportion of variance explained by the fixed effects. Conditional R^2^: proportion of variance explained by the model. Significant *p*-values (*p<*0.05) are shown in bold.

## Discussion

Path integration is the process by which animals update their internal representation of their position by integrating their movement (linear and angular displacement) from a starting point^72,73^. It is thought to be the process many animals use for homing (the ability to return home, e.g., after foraging). Optic flow, the visual stimulus produced while navigating the environment, has been shown to be critical for path integration in several species, from insects to primates^5–7,34,74–79^. In ants, odometry has been shown to rely on visual and nonvisual cues, such as stride-length^39,40^. In humans, even though studies have shown that vestibular information^80^ and “body senses” (a combination of vestibular and proprioceptive information)^43,81^ might be equally or even more important than visual information for path integration, it has been shown that optic flow information is also sufficient for estimating distances^5^.

In the absence of any stimulus other than a visual self-motion stimulus, i.e., no vestibular information or stride-length information, we hypothesized that participants would try to use all possible types of information that were available to them. Optic flow elicits characteristic, optokinetic-like eye movements (slow eye movements following the motion of the stimulus near the fovea^12^ and rapid (saccadic) eye movements^13–15^). Therefore, theoretically, if participants integrate the movement trajectory of their own eyes while tracking the visual self-motion stimulus, they can extract reliable information about the travel distance (which we term oculomotor odometry).

In this study, we explored according to the idea of an oculomotor odometer, the role of eye movements on human distance perception from visually simulated self-motion. We expected our fixation instruction to be detrimental to the perception of self-motion distances. To our surprise, we found no significant differences in the PSE, the point at which both distances were perceived as equal, between our fixation conditions. Nonetheless, fixation conditions modulated participants’ overall response bias, where Fix01 led observers to a significantly lower proportion of responses “2nd distance is longer”, as seen by our GLMM results. We see a slight opposite effect for Fix10, but it remains not significant. We believe our results are in line with a possible oculomotor odometer, even though the importance that participants give to this particular signal is lower than hypothesized. All in all, this indicates that fixation, compared with free gaze, does not strongly impact distance perception. Our findings suggest that participants are able to compare travel distances better if they can rely on consistent eye-movement patterns across both self-motion epochs compared with situations where one epoch involves fixation and the other involves free-viewing. This aligns with previous work^14^, showing that participants’ eye movements during the reproduction of a traveled distance closely mirror those during the initial encoding phase, with eye position and velocity showing trial-by-trial correlations.

The eye control windows employed in our study were large compared to other studies on visual perception during fixation. They had been chosen because of unavoidable residual eye movements during fixation and the presentation of large field visual motion. These residual eye movements create a potential confound towards the goal of our study. Yet we already considered this by our analyses. As an example, depicted in Fig. 4, we compared the real eye movements during the free viewing and fixation conditions, and did not assume that eyes were perfectly still in the fixation condition. Importantly, our approach was chosen also in line with previous work^82^.

Chow et al.^82^ reported that in some “easier” tasks of direction perception, the restriction of eye movements also did not influence behavior, but more challenging tasks did. It could have been that our task was too easy for the participants to require larger eye movements for good performance. However, given the accuracy of approximately 67% for our smallest differences in travel distances (distance ratios of 0.875 and 1.125) and that participants did not reach 100% accuracy consistently for our largest differences (distance ratios of 0.5 and 1.5), we argue that our task was indeed challenging.

Churan et al.^14^ reported that when participants conducted a distance reproduction task, they preferred to direct their eyes mostly to a small area on the screen, referred to as the preferred eye position (PEP). The PEP’s position and width seem to vary between participants, but the area bound by one standard deviation of a 2D Gaussian fit on their eye position showed that most participants move their eyes only in an area of approximately 1.5–3 deg^2^ (Churan 2018). This area is smaller than our fixation window restriction of 2° around the fixation dot. Even when the PEPs shown in Churan et al. were found to be mostly on the horizon and lower, it is possible that our fixation window only shifted the central position of this distribution upward. Given that the fixation window contains, and the center is very close to, the FOE, it is possible that we did not restrict eye movements as much as needed to disrupt behavior.

It could be argued that participants might have used temporal information rather than oculomotor odometry to solve the task. However, in our experiment, speeds and distances were chosen randomly, not for allowing, e.g., to simply count duration in seconds for judging which of the two distances were longer. Furthermore, in a previous study employing a similar paradigm (with fixation required during both displacements), we could unequivocally rule out temporal information as the basis for task solving^7^.

To conclude, our results suggest that making participants fixate during a distance perception (or reproduction) paradigm does not largely interfere with behavior and can be used to avoid issues such as EEG artifacts arising from eye movements, as long as the fixation (or lack thereof) remains consistent throughout the experiment.

## Supplementary Information

Below is the link to the electronic supplementary material.

Supplementary Material 1

## Data Availability

Raw and aggregated data, and stimulus and analysis scripts can be found at https://tam-datahub.online.uni-marburg.de/handle/tam/43.
