# Supplementary material for: Path integration from optic flow and the role of eye movements
Source: Sci Rep. 2026 Jun 7;16:17540. doi: 10.1038/s41598-026-56170-9 (PMC13243615; doi:10.1038/s41598-026-56170-9)
Supplement: Supplementary file 1 — Supplementary Material 1 [file 41598_2026_56170_MOESM1_ESM.docx]

### **Supplementary materials**

### **Supplementary methods:**

### **Validation of psychometric parameters via lapse-rate modeling**

To address potential stimulus-independent errors (lapses), we implemented a hierarchical, non-linear Bayesian model using the *brms* [60] package in R. The probability of a response was modeled as: *P(Answer) = λ + (1 - 2 λ) × logit^-1^(η)* where *η* is the linear predictor for the experimental conditions (fixation condition, z-scored distance ratio) and *λ* represents the lapse rate. To ensure model convergence and realistic parameter estimation, we applied a prior on the logit-transformed lapse-rate (*logit(λ) ~ N(-4.6,0.25)*), corresponding to a prior expectation of approximately a 1% lapse rate. Individual lapse rates are shown in Supplementary Figure 2.

Supplementary tables 1 and 2 show the PSE and JND for the GLMM and the Bayesian models, respectively. The high correlation (PSE: Pearson’s *R* = 0.999, *p* = 0.001; JND: Pearson’s *R* = 0.973, *p* = 0.027) and overlapping confidence and credible intervals suggest that while individual lapses were present, they did not systematically change the results reported in the main text.

| Fixation | PSE | PSE Lower CI | PSE Upper CI | JND | JND Lower CI | JND Upper CI |
| --- | --- | --- | --- | --- | --- | --- |
| Fix00 | 0.9942 | 0.9435 | 1.0449 | 0.1723 | 0.1397 | 0.2049 |
| Fix01 | 1.0718 | 1.0236 | 1.1200 | 0.1696 | 0.1378 | 0.2013 |
| Fix10 | 0.9795 | 0.9236 | 1.0354 | 0.1889 | 0.1508 | 0.2270 |
| Fix11 | 1.0259 | 0.9785 | 1.0732 | 0.1636 | 0.1337 | 0.1934 |

**Supplementary Table 1. GLMM Results (CI = 95% Confidence Interval).**

| Fixation | PSE | PSE Lower CI | PSE Upper CI | JND | JND Lower CI | JND Upper CI |
| --- | --- | --- | --- | --- | --- | --- |
| Fix00 | 0.9868 | 0.9234 | 1.0476 | 0.1539 | 0.1189 | 0.1989 |
| Fix01 | 1.0666 | 1.0070 | 1.1310 | 0.1557 | 0.1222 | 0.1995 |
| Fix10 | 0.9768 | 0.9032 | 1.0459 | 0.1761 | 0.1366 | 0.2293 |
| Fix11 | 1.0207 | 0.9602 | 1.0816 | 0.1509 | 0.1197 | 0.1926 |

**Supplementary Table 2. Non-Linear Lapse Model Results (CI = 95% Credible Interval).**

### **Supplementary figures:**

**
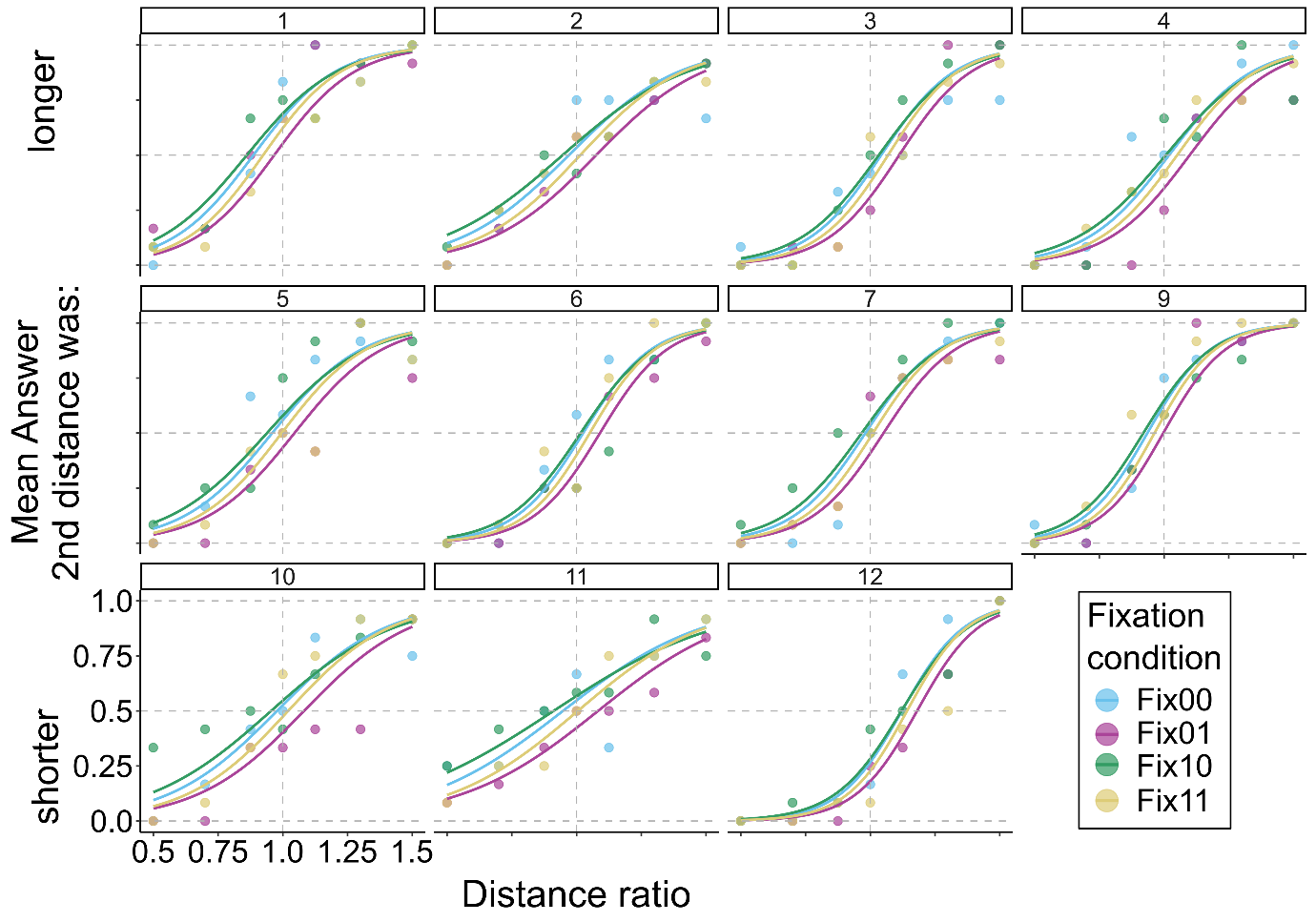
 Supplementary Figure 1. Generalized linear mixed-effects model predictions for individual participants.** The mean proportion of “2^nd^ distance was longer” responses for each fixation condition (Fix00: blue, Fix01: pink, Fix10: green, Fix11: yellow) are shown as a function of the distance ratio (filled dots), superimposed with the model-predicted psychometric functions (solid lines). Each panel shows the result for one of the eleven participants.


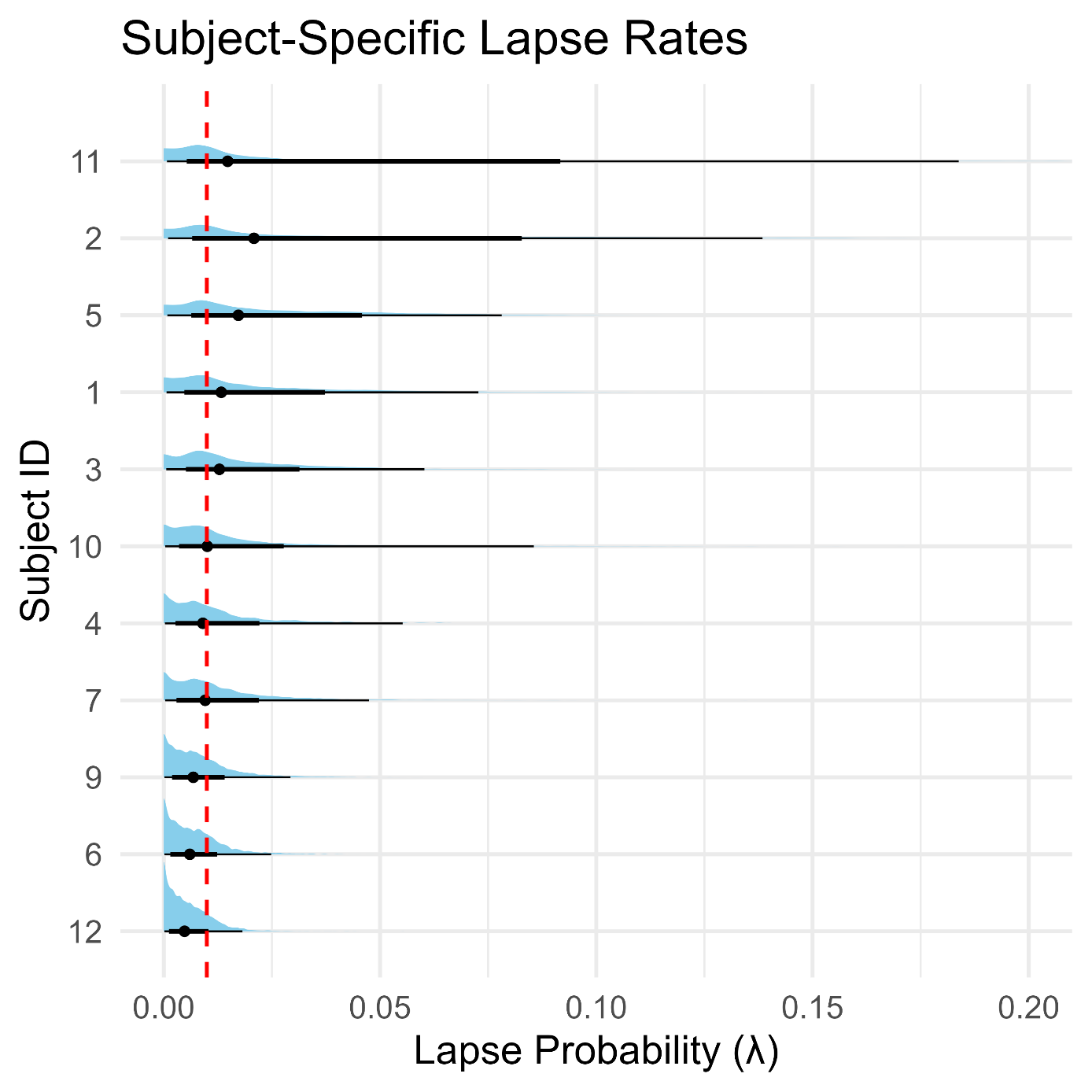


**Supplementary Figure 2. Individualized probability of lapses.** Bayesian posterior distributions (in light blue) for each participant (different rows) as a function of lapse probability (a value of 0.01 indicates that on any given trial, there was a 1% chance the participant answered without looking at the stimulus). Black dots represent the median of the posterior distribution; the thick horizontal lines show the 66% Credible Interval and the thin lines indicate the 95% Credible Interval. The vertical red dashed line shows the center of our prior distribution (at roughly 1%).
